# Aesthetic Rehabilitation in Teeth with Wear from Bruxism and Acid Erosion

**DOI:** 10.2174/1874210601812010486

**Published:** 2018-07-31

**Authors:** Pamella Tomazi Godoy de Oliveira, Deise Caren Somacal, Luiz Henrique Burnett Júnior, Ana Maria Spohr

**Affiliations:** Department of Restorative Dentistry, School of Dentistry, Pontifical Catholic University of Rio Grande do Sul, Porto Alegre, Brazil

**Keywords:** Bruxism, Dental erosion, Ceramic laminates, Oral rehabilitation, Gingivoplasty, Fracture of restorations

## Abstract

**Background:**

Bruxism is defined as a repetitive activity of grinding the teeth through lateral or protrusive movements of the mandible, and it is considered the most complex and destructive functional disorder. In addition, erosion caused by diet or reflux can damage the remaining teeth.

**Objective:**

In this report, a patient with bruxism and dental erosion was treated with a multidisciplinary approach to restore the function and aesthetic.

**Methods:**

This clinical report describes the management of an adult woman, 33 years old, who was dissatisfied with the aesthetics of her smile and complained of joint pain and headaches. As result of her condition, the patient’s dentition exhibited generalized wear on the vestibular and incisal surface of the upper incisors incompatible with her age, moderate darkening of the teeth and excess gingival tissue in the upper incisors. After a detailed anamnesis and clinical examination, a diagnosis of bruxism and acid erosion caused by a diet rich in citrus foods and beverages was obtained. Forthwith, a treatment plan was established, and the patient underwent home bleaching, gingivoplasty and ceramic laminates of lithium disilicate on the anterior teeth. After the rehabilitation was completed, a night guard was made to reduce the symptoms of bruxism and avoid fracture of the ceramic restorations. The patient was followed at different time intervals.

**Conclusion:**

The improvement in the aesthetics of the teeth was significant and remained stable after periodic controls in which no adverse effects were observed.

## 1 INTRODUCTION

Bruxism is defined as a repetitive activity of grinding the teeth through lateral or protrusive movements of the mandible [1, 2]. It is a habit that causes great concern among dental surgeons because of its consequences: tooth destruction, fracture of restorations or necessity of prosthetic rehabilitation [3]. At this time, signs and symptoms such as exacerbation of temporomandibular disorders, induction of temporal headache and tooth-grinding sounds that may interfere with the family’s sleep are also perceptible [4].

Bruxism is commonly seen in the population and it can occur in all age groups, with a prevalence in both genders and a presence in 31% of adults [5]. It is considered the most complex and destructive functional tooth disorder, which requires a multidisciplinary approach to obtain the best prognosis for the patient and its treatment. In some cases, this parafunctional habit may threaten the integrity of the stomatognathic system structures if the magnitude and direction of the forces exerted exceed the adaptive capacity. The multifactorial etiology of orofacial pain and temporomandibular disorders makes it difficult for the dentist to treat them alone, even when bruxism is involved in the etiology [6].

One of the main challenges of dentistry is to identify whether the patient presents bruxism in the daytime or nighttime according to his report of myofascial pain [4]. Sleep bruxism is a common chronic condition in approximately 8% of the population [7] and, if improperly diagnosed or untreated, it can cause considerable damage to the teeth; the repair is complex [8]. According to a recent review, both types of bruxism, both physiological and pathological, have associated causal factors that are yet unknown. However, some conditions, such as smoking, medication use and respiratory problems may be considered risk factors for bruxism [4].

Nevertheless, other congenital anomalies such as parafunctional habits, abrasion, and erosion caused by diet or reflux can damage the remaining teeth. The nature of dental erosion is related to the presence of non-bacterial acids in the oral cavity. Acids may come from extrinsic sources, such as food and beverages, or intrinsic sources, due to the presence of gastric acid (hydrochloric acid) [9]. Diet plays an important role [10]. Frequency of consumption [11], time of contact with acid [12] and unusual patterns of consumption are also relevant factors that influence the erosive effect. In patients with teeth eroded and worn by bruxism, the main cause of tooth structure loss should be determined to eliminate etiologic factors before rehabilitation therapy is performed [13].

Thus, regardless of the etiology, tooth wear is considered pathological when the loss of tooth structure is not compatible with the patient's age, requiring alternative rehabilitation treatment to restore the lost dental shape and aesthetics.

The aim is to present a case report of a patient with dental erosion caused by bruxism and acid erosion, who was treated with indirect and minimally invasive techniques for aesthetic and functional smile rehabilitation.

## 2 CASE REPORT

A 33-year-old female patient visited the Faculty of Dentistry Clinic of the Pontifical Catholic University of Rio Grande do Sul, in Porto Alegre City, Brazil, because she was dissatisfied with the aesthetics of her smile and felt uncomfortable with the appearance of her two upper central incisors and two upper lateral incisors.

**Fig. (1):**
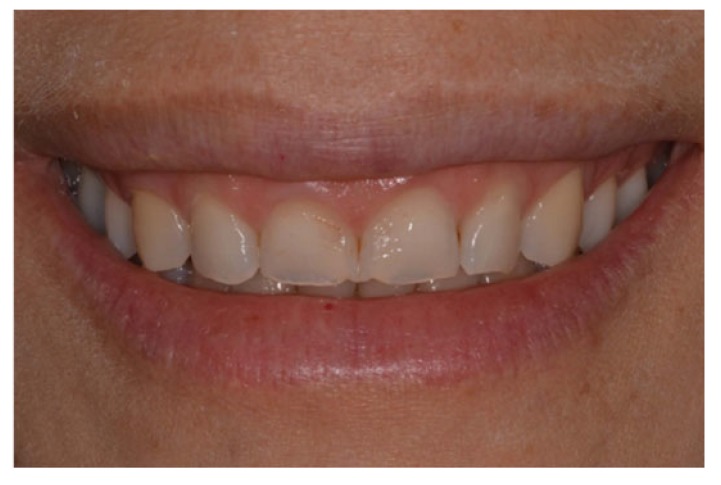
Severe wear of the four upper incisors.

In the anamnesis, the patient reported that she had pain in the cervical region, a feeling of fatigue in the face, and the presence of tooth wear incompatible with her age; there was also an emotional component because she did not like her smile. The patient also reported that she did not use any bruxism night guard. During the clinical examination, moderate dental dimming, the presence of acid erosion on the buccal surfaces of the four upper incisors, and severe wear on the incisal edge region of the same teeth were observed (Fig. **1**).

After a careful clinical evaluation and radiographic examination, and according to the patient’s main complaint, functional and aesthetic reestablishment were planned as follow: dental bleaching of both arches; gingivoplasty of upper right central incisor, upper left central incisor, upper right canine, and upper left canine (upper right and left lateral incisors did not undergo surgical intervention); ceramic laminates for upper central incisors and upper lateral incisors; and a bruxism night guard.

**Fig. (2):**
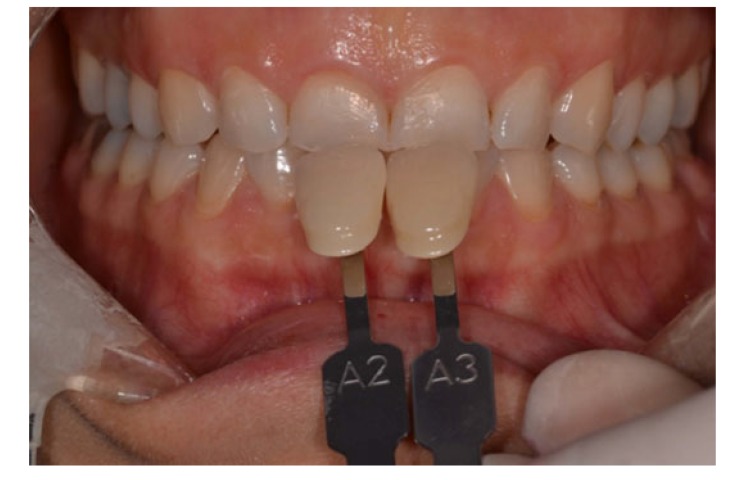
Color registration before tooth whitening.

After the patient agreed with the proposed treatment, the external bleaching was performed using a homemade technique with trays and 7.5% hydrogen peroxide (White Class / FGM Dental Products, Joinville, SC, Brazil). Initially, the tooth color was recorded using the Vita scale (Vita Toothguide, Zahnfabrick, Bad Sackingen, Germany), which was defined as A2 in the incisor region and A3 in the canine region (Fig. **2**).

**Fig. (3):**
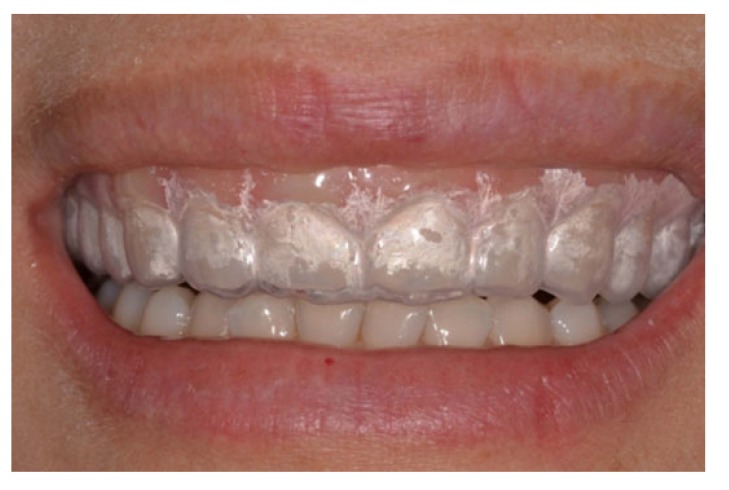
Bleaching tray positioned on the upper teeth.

The patient was instructed to use the silicone trays made in a thermo-vacuum apparatus with 7.5% hydrogen peroxide gel, one hour per day, for four weeks (Fig. **3**).

The gel was applied inside of the tray corresponding to the buccal face of the upper and lower teeth. After completion of the bleaching, a color registration was performed and a reduction of the tooth saturation level was observed. The color B1 was defined in the selection phase for the lithium disilicate ceramic restorations (IPS e.max, Ivoclar Vivadent, Schaan, Liechtenstein).

**Fig. (4):**
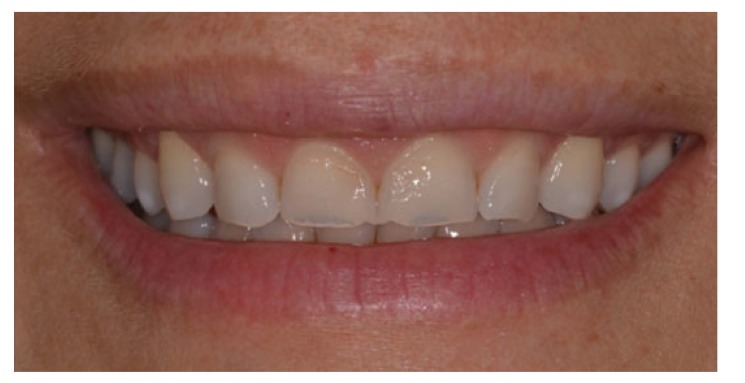
Postoperative 7-day gingivoplasty.

A zirconia bur (Tissue Trimmer, NTI, Kahla, Thuringia, Germany) was used to perform the gingivoplasty on upper right central incisor, upper left central incisor, upper right canine, and upper left canine; its function was to remove excess gum tissue through immediate cauterization and cutting, without requiring any type of suture. The spear-shaped zirconia tip was positioned perpendicularly to the long axis of the tooth, and the gingivoplasty was performed carefully and delicately. The seven-day postoperative period showed well-healed tissue, with no sign of edema, redness, or any inflammatory signal (Fig. **4**).

After 30 days, two impressions were obtained with irreversible hydrocolloid (Hydrogum, Zhermack, Rome, Italy) to perform diagnostic waxing. In the same session, initial photographs were taken to communicate with the laboratory and determine the final format of the teeth to be restored. The waxing model lets the dentist obtain the final anatomy reference of the teeth to be restored. Still in the preliminary laboratory phase, a guide was prepared with a vinyl polysiloxane impression material (Variotime, Heraeus Kulzer, Hanau, Hesse, Germany), which was used to guide the preparation of both the laminates and the provisionals with bis-acryl resin (Protemp, 3M-ESPE, Saint Paul, MN, USA).

**Fig. (5):**
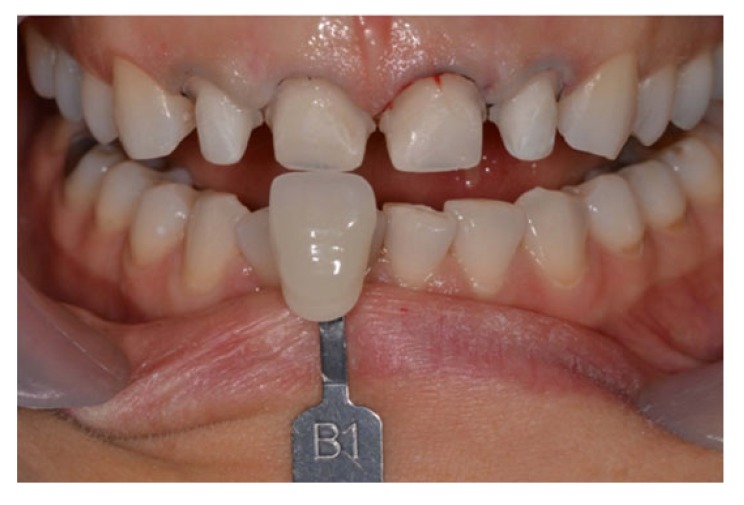
Prepared teeth and color recording of the dental substrate.

Before beginning preparation for the four upper incisors, # 000 and #0 gingival retraction cords (Ultradent, Oraltech, Indaiatuba, SP, Brazil) were used to protect the gingival sulcus with a hemostatic solution (Hemostop, Dentsply, York, USA). Initially, the teeth wear was performed using a coarse chamfer diamond bur # 4138 (Kg Sorensen, Cotia, SP, Brazil) and completed with the same diameter tips of medium grit. Next,1.5 mm incisal wear at 45˚ was performed and completed with medium-sized aluminum oxide pop-on discs (Sof-lex, 3M-ESPE, Saint Paul, MN, USA) (Fig. **5**).

**Fig. (6):**
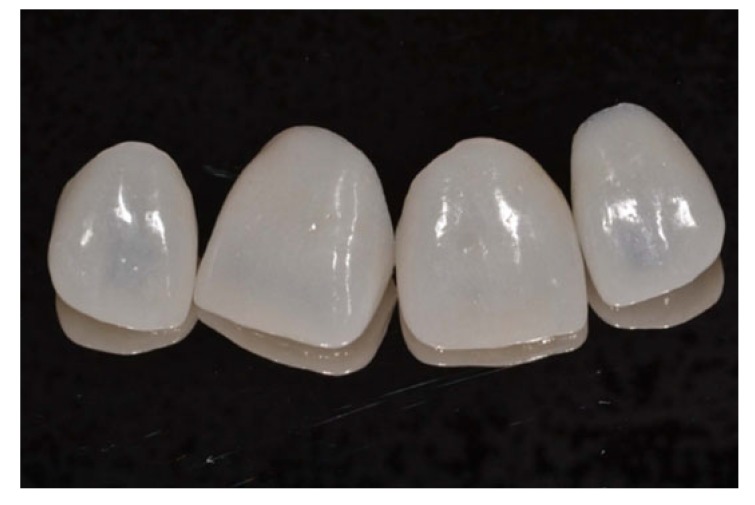
Laminates with lithium disilicate ceramic (IPS e.max).

In the same session, the simultaneous double impression technique was performed in which the second gingival retraction cord # 0 was removed, enabling the first cord # 000 to maintain the gingival sulcus hemostasis. The impression was performed with a vinyl polysiloxane impression material (Variotime). In the sequence, the provisionals were adjusted and fixed through a silicone guide and bis-acryl resin. The ceramic material chosen was lithium disilicate (IPS e-max, Ivoclar-Vivadent, Schaan, Liechtenstein) (Fig. **6**).

After the removal of the provisionals, the restorations were placed on the prepared teeth to evaluate the marginal adaptation of the restorations, and after the patient’s approval, the final cementation of the restorations with resin cement shade A1 was performed (RelyX Veneer, 3M-ESPE, Saint Paul, MN, USA).

**Fig. (7):**
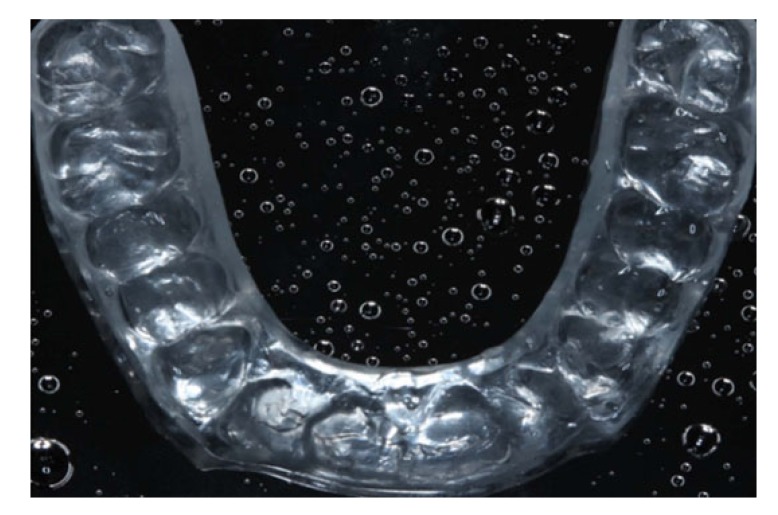
Bruxism night guard.

After the occlusal adjustment, an irreversible hydrocolloid impression was performed to produce a hard acrylic night guard (Fig. **7**), following the Michigan Protocol. The guard was made as soon as possible to avoid the occurrence of injuries to the restorations, such as cracks, splinters in the incisal edge or even fractures. After the device was made, the occlusal adjustment was performed in left/right laterality, protrusion and Maximum Habitual Intercuspation (MIH).

**Fig. (8):**
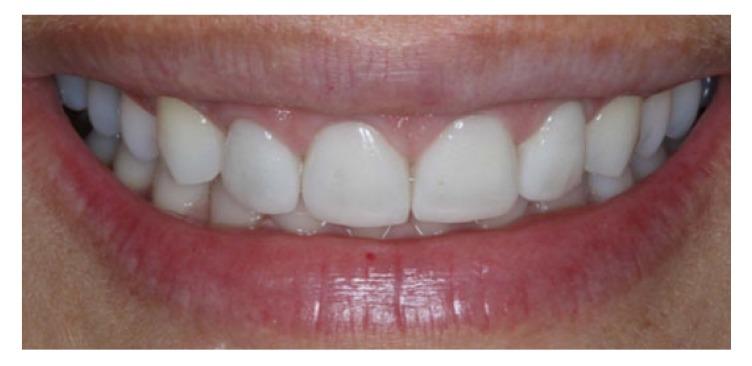
Completed restorative treatment.

Periodic maintenance appointments were scheduled every six months, which included prophylaxis and revision of the night guard. The gingival tissue remained intact, with no inflammatory signal at the probe, and the visual examination revealed a light pink and orange peel appearance, typical of healthy gingival tissue (Fig. **8**).

## 3 DISCUSSION

Bruxism is defined as a temporomandibular disorder, with diurnal (centric bruxism) or nocturnal (eccentric bruxism) manifestations [2]. Daytime bruxism is characterized by a semi-voluntary activity of the jaw and clenching of the teeth while the person is awake; the grinding of teeth generally does not occur. Daytime bruxism is related to a vicious habit, such as the act of biting a pencil, a pen or other objects, and is characterized as centric bruxism [14]. Whereas eccentric bruxism is the unconscious activity of grinding or squeezing and sliding the teeth into the protrusive and latero-protrusive positions, producing sounds while the person is sleeping [2].

The result of the centric or eccentric bruxism is observed by wearing of the dental structure. When the patient presents wear of the anterior teeth, there is a loss of anterior guide protection and the presence of posterior interferences [15]. Therefore, in the dental treatment for these cases, the most suitable restorative material should be evaluated, considering the patient occlusion and the need for incisal/occlusal reconstruction. The restorative material must have aesthetic and resistance properties similar to tooth enamel [16]. In the present report, the use of ceramic laminates was justified by the high resistance to wear, compression and excellence in mimicking dental structures, in addition to allowing conservative treatment [16].

In cosmetic dentistry, one of the most requested procedures is tooth whitening, as it is a safe, conservative and simple procedure [17]. It is common to perform it before rehabilitating the patient with ceramic laminates, aiming at color stability and patient satisfaction.

Acid erosion is the loss of tooth structure by chemical action in the continuous presence of demineralizing agents. Patients with constant eating habits such as the ingestion of citrus fruits, contact with chlorinated pool water or gastrointestinal problems that produce a repeated exposure of the teeth to hydrochloric acid present in gastric juice may develop acid erosion. Considering that the pH of citric acid is 2.5 and the critical point at which enamel dissolves is the pH range of 5.0-5.7, citric acid may play a significant role in tooth erosion [13]. It is therefore relevant to diagnose acid erosion at the anamnesis and clinical examination to institute better treatment before the aesthetic rehabilitation.

A bite plaque is essential for protecting the teeth from erosion and maintaining the vertical dimension of occlusion through the mutually protected occlusion [13]. The occlusal bite guard is made after the completion of the treatment and is designed for continuous use.

The present case demonstrates an association of bruxism and acid erosion in the etiology of tooth wear. It is relevant to diagnose such changes and institute the appropriate therapy, including not only aesthetic rehabilitation but also the correct treatment of the causes of wear. The combination of treatments makes the reported case complex, yet common in dental clinical practice. Longitudinal clinical studies are still necessary to achieve the use of minimally invasive dentistry for the treatment of bruxism and aesthetic erosion.

## CONCLUSION

The wear of the upper incisors caused by bruxism and acid erosion were restored with lithium disilicate laminates after bleaching and gingivoplasty. At the end of the treatment, the patient’s aesthetic and functional requirements were renewed.

## References

[1] Tago C., Aoki S., Sato S. (2017). Status of occlusal contact during sleep bruxism in patients who visited dental clinics: A study using a Bruxchecker^®^.. Cranio.

[2] Lobbezoo F., Ahlberg J., Glaros A.G., Kato T., Koyano K., Lavigne G.J., de Leeuw R., Manfredini D., Svensson P., Winocur E. (2013). Bruxism defined and graded: An international consensus.. J. Oral Rehabil..

[3] Onodera K., Kawagoe T., Sasaguri K., Protacio-Quismundo C., Sato S. (2006). The use of a bruxchecker in the evaluation of different grinding patterns during sleep bruxism.. Cranio.

[4] Lavigne G.J., Khoury S., Abe S., Yamaguchi T., Raphael K. (2008). Bruxism physiology and pathology: An overview for clinicians.. J. Oral Rehabil..

[5] Mesko M.E., Hutton B., Skupien J.A., Sarkis-Onofre R., Moher D., Pereira-Cenci T. (2017). Therapies for bruxism: A systematic review and network meta-analysis (protocol).. Syst. Rev..

[6] de la Hoz-Aizpurua J.L., Díaz-Alonso E., LaTouche-Arbizu R., Mesa-Jiménez J. (2011). Sleep bruxism. Conceptual review and update.. Med. Oral Patol. Oral Cir. Bucal.

[7] Hirai K., Ikawa T., Shigeta Y., Shigemoto S., Ogawa T. (2017). Evaluation of sleep bruxism with a novel designed occlusal splint.. J. Prosthodont. Res..

[8] McAuliffe P., Kim J.H., Diamond D., Lau K.T., O’Connell B.C. (2015). A sleep bruxism detection system based on sensors in a splint - Pilot clinical data.. J. Oral Rehabil..

[9] Salas M.M., Nascimento G.G., Vargas-Ferreira F., Tarquinio S.B., Huysmans M.C., Demarco F.F. (2015). Diet influenced tooth erosion prevalence in children and adolescents: Results of a meta-analysis and meta-regression.. J. Dent..

[10] Al-Dlaigan Y.H., Shaw L., Smith A. (2001). Dental erosion in a group of British 14-year-old school children. Part II: Influence of dietary intake.. Br. Dent. J..

[11] Aidi H.E., Bronkhorst E.M., Huysmans M.C., Truin G.J. (2011). Factors associated with the incidence of erosive wear in upper incisors and lower first molars: A multifactorial approach.. J. Dent..

[12] van Rijkom H.M., Truin G.J., Frencken J.E., König K.G., van ’t Hof M.A., Bronkhorst E.M., Roeters F.J. (2002). Prevalence, distribution and background variables of smooth-bordered tooth wear in teenagers in the hague, the Netherlands.. Caries Res..

[13] Guldag M.U., Buyukkaplan U.S., Ay Z.Y., Katirci G. (2008). A multidisciplinary approach to dental erosion: A case report.. Eur. J. Dent..

[14] Goldstein R.E., Auclair Clark W. (2017). The clinical management of awake bruxism.. J. Am. Dent. Assoc..

[15] Doan P.D., Goldstein G.R. (2007). The use of a diagnostic matrix in the management of the severely worn dentition.. J. Prosthodont..

[16] Granell-Ruíz M., Agustín-Panadero R., Fons-Font A., Román-Rodríguez J.L., Solá-Ruíz M.F. (2014). Influence of bruxism on survival of porcelain laminate veneers.. Med. Oral Patol. Oral Cir. Bucal.

[17] De Geus J.L., Wambier L.M., Kossatz S. (2016). At-home vc in-office bleaching: A systematic review and meta-analysis.. Oper Dent.

